# Simple, sensitive, specific self-sampling assay secures SARS-CoV-2 antibody signals in sero-prevalence and post-vaccine studies

**DOI:** 10.1038/s41598-022-05640-x

**Published:** 2022-02-03

**Authors:** Maryam Khan, Carolina Rosadas, Ksenia Katsanovskaja, Isaac D. Weber, Justin Shute, Samreen Ijaz, Federica Marchesin, Eleanor McClure, Salem Elias, Barnaby Flower, He Gao, Rachael Quinlan, Charlotte Short, Annachiara Rosa, Chloe Roustan, Maya Moshe, Graham P. Taylor, Paul Elliott, Graham S. Cooke, Peter Cherepanov, Eleanor Parker, Myra O. McClure, Richard S. Tedder

**Affiliations:** 1 Department of Infectious Disease, Faculty of Medicine, Imperial College London, St Mary’s Campus, Praed Street, London, W2 1NY UK; 2 Public Health England, 61 Colindale Ave, London, NW9 5EQ UK; 3 Francis Crick Institute, 1 Midland Rd, Somers Town, London, NW1 1AT UK; 4 Imperial College Healthcare NHS Trust, St Mary’s Hospital, Praed St, Paddington, London, W2 1NY UK; 5 Department of Epidemiology and Biostatistics, School of Public Health, MRC Centre for Environment and Health, Imperial College London, London, UK; 6 NIHR Imperial Biomedical Research Centre, Imperial College London, Exhibition Rd, London, SW7 2AZ UK

**Keywords:** SARS-CoV-2, RNA vaccines, Acute inflammation, Viral infection

## Abstract

At-home sampling is key to large scale seroprevalence studies. Dried blood spot (DBS) self-sampling removes the need for medical personnel for specimen collection but facilitates specimen referral to an appropriately accredited laboratory for accurate sample analysis. To establish a highly sensitive and specific antibody assay that would facilitate self-sampling for prevalence and vaccine-response studies. Paired sera and DBS eluates collected from 439 sero-positive, 382 sero-negative individuals and DBS from 34 vaccine recipients were assayed by capture ELISAs for IgG and IgM antibody to SARS-CoV-2. IgG and IgM combined on DBS eluates achieved a diagnostic sensitivity of 97.9% (95%CI 96.6 to 99.3) and a specificity of 99.2% (95% CI 98.4 to 100) compared to serum, displaying limits of detection equivalent to 23 and 10 WHO IU/ml, respectively. A strong correlation (r = 0.81) was observed between serum and DBS reactivities. Reactivity remained stable with samples deliberately rendered inadequate, (*p* = 0.234) and when samples were accidentally damaged or ‘invalid’. All vaccine recipients were sero-positive. This assay provides a secure method for self-sampling by DBS with a sensitivity comparable to serum. The feasibility of DBS testing in sero-prevalence studies and in monitoring post-vaccine responses was confirmed, offering a robust and reliable tool for serological monitoring at a population level.

## Introduction

The detection of antibodies against SARS-CoV-2 is central to sero-prevalence studies^1–3^. Large scale sero-prevalence studies reliant on serum testing are expensive and limited by the logistics of blood taking and transport to central laboratories^4^. As an alternative, national sero-prevalence studies have been undertaken using self-administered lateral flow testing^5,6^. However, while the lateral flow tests used to date have been evaluated for population studies, their performance does not yet meet those demanded by UK regulators for home testing for clinical purposes and they are, by definition, not amenable to quality assurance.

Dried blood spots (DBS) offer an ideal platform for large-scale sero-prevalence studies and have been extensively used for other blood borne viruses^7,8^. Detection of antibody can be undertaken in assays of different formats, each of which brings particular attributes to the testing^9^. The recognition that other body fluids, including eluates from DBS, offer alternatives for analysis has led to the adoption of immunoglobulin (Ig) capture assays^10,11^.

Capture assays are not susceptible to the disturbance of signal caused by fluctuation in Ig levels which so adversely affect both the indirect immunoassays, signalling by labelled antibody to human Ig, and the double antigen binding assay (DABA) format. This susceptibility has been shown, predictably, to be the case for seeking antibody to SARS-CoV-2 with the Roche anti-NP DABA^12^. In a meticulously detailed study which also exemplified the variability of DBS sampling, the authors demonstrated that the sensitivity afforded by coupling DBS to the Roche assay was inadequate. This was the result of their choice to use an assay format inherently sensitive to the concentration of plasma in the diluent, in their estimate being at best only 10% plasma. However, the inference drawn by the authors that DBS sampling cannot inform on the sero-epidemiology of SARS-CoV-2 through a lack of sensitivity is simply wrong, as we show below. We believe correction of this misleading assertion requires address.

Here we describe the extensive use, characterisation and robustness of an immunoglobulin capture assay to detect antibody from DBS eluates and compare its sensitivity and specificity to the Imperial hybrid DABA using serum samples^13,14^. As of yet, no published study describing antibody responses using DBS has validated their methodology on an adequate number of samples, or has been successful in using Ig capture assays for detection of antibody from DBS^15–17^. We demonstrate a very high sensitivity and specificity of a capture assay for detecting anti-SARS-CoV-2 by DBS sampling from a large cohort of persons known to be seropositive following recovery from Covid-19 illness. These data show clearly that there remains an important role for DBS sampling in conducting sero-prevalence studies in both the UK and elsewhere^18^, especially where venesection and transport of fluid blood samples is not easily delivered. In addition, the use of DBS sampling avoids the need to handle other non-invasive but potentially infectious bodily fluids, such as oral fluid, and facilitates the use of postal systems for safe sample transport. Further, we demonstrate DBS sampling coupled with Ig capture assays opens up the potential for monitoring vaccine responsiveness on a national scale.

## Methods

### Sample collection and processing

Paired serum and DBS samples were collected from 439 individuals recovered from SARS-CoV-2 infection, confirmed by RT-PCR in all those so tested. Patients were representative of the full range of Covid 19 symptoms and were at least 14 days post-onset of symptoms at the time of sampling. Seropositivity was confirmed by the Imperial Hybrid DABA detecting antibody to the receptor binding domain (anti-RBD)^13,14^. Antibody negative matched serum and DBS samples from 382 individuals so determined by the anti-RBD DABA assay were used to determine the specificity of DBS testing on an S1 Ig capture assay. DBS samples were collected and processed as described below and serum samples were obtained through venepuncture, centrifuged at 800*g* and stored at − 80 °C until ready for assaying. DBS samples were collected from vaccine recipients at least 14 days post immunisation.

Dried blood spots were collected at home by participants via self-sampling. All DBS samples were collected on AHLSTROM MUNKSJO BioSample TFN 12 mm cards designed for screening infections^19,20^.These cards are made from absorbent fibres without the addition of wet-strength additives or chemicals^19^. Briefly, participants are invited to disinfect their hands and prick the side of their finger with a lancet. The blood droplet is formed by gentle squeezing of the finger, placed above the DBS card and allowed to drop on to the card while avoiding smearing. Once added to the first circle, blood drops are added to the remaining circles. The DBS cards were considered valid for testing if the blood had soaked through and was visible on the reverse side of the card. The DBS cards were then air-dried in an upright position to avoid surface contamination and transported for testing in a sample sleeve to the laboratory where they were stored at 5 °C for 1 week, or for longer at -20 °C to ensure sample stability. Inoculated cards were brought to room temperature before processing. The elution procedure was performed in Salivette® tubes (Sarstedt, Nümbrecht, Germany) which, prior to the elution, were filled with 250 µl of previously prepared elution buffer (Phosphate buffered saline, PBS, pH 7.4, supplemented with 1% volume sodium azide (8% solution), 0.05% Tween-20 and 2% rabbit serum (Sigma Aldrich). The pre-perforated DBS spot was pressed out of the card and dropped into the solution. To ensure a complete DBS spot submersion into the elution buffer, the Salivette® tubes with the samples were briefly vortexed and incubated overnight at 4^0^C. Following the incubation, the Salivette tubes were centrifuged at 4110*g* for 5 min. The resulting eluate was collected into a Sarstedt tube and analysed or stored at 4 °C.

### Antigens

All proteins used were produced at The Francis Crick Institute. The SARS-CoV-2 RBD and S1 constructs, spanning SARS-CoV-2 S (NCBI reference NC_045512) residues 319-541 (RVQPT…KCVNF) and 1-530 (MFVFL…GPKKS), respectively are produced with C-terminal twin Strep tags, cloned into mammalian expression vector, pQ-3C-2xStrep (PubMed ID 31907454). Transfection of Expi293F cells with the corresponding plasmids was carried out using ExpiFectamine (Thermo Fisher Scientific, Massachusetts, US) and proteins purified to homogeneity by size-exclusion chromatography (Superdex 200, GE Healthcare). S1 and RBD antigens were conjugated to horseradish peroxidase (HRP) using Bio-Rad LYNX HRP conjugation kit, as per manufacturer’s instructions and used, respectively, as revealing agents in the Ig capture assays and in the Imperial Hybrid DABA.

### Enzyme immunoassays

#### Anti-SARS-CoV-2 S1 IgG and IgM capture ELISAs

Microwells were coated with 100 μl of either 5 µg/ml rabbit anti-human IgG (Stratech Scientific, Ely, UK) or 2.5 µg/ml anti-human IgM (Stratech Scientific, Ely, UK) in coating buffer (Clintech, Guildford, UK) and incubated overnight at 2 to 8 °C. Wells were washed with PBS/0.05% Tween-20 once and blocked using 200 µl/well blocking solution (Microimmune, Guildford, UK) before drying overnight at 37 °C.

One hundred microlitres of either eluted DBS or sera pre-diluted at 1:100 in sample buffer PBS Tween 0.05%, Gentamicin 0.5% and Amphotericin 0.2% supplemented with 10% fetal calf serum) were added to the coated plates and incubated for 1 h at 37 °C. Plates were washed five times (Wash buffer, Clin-Tech), followed by the addition of 100 µl of SARS-CoV-2 S1 conjugated with HRP appropriately diluted in conjugate buffer (Clin-Tech). After further incubation for 1 h at 37 °C, the plate was washed five times and 100 µl TMB substrate (Clin-Tech) added to each well, followed by a further 30 min incubation at 37 °C. Reactions were stopped by the addition of 50 µl/well 0.5 M sulphuric acid (Microimmune). Optical densities (ODs) were measured by SpectraMax M2 (Molecular Devices, San Jose, CA, USA) at 450/630 nm. A cut-off value was calculated for each run (average OD of the negative-control triplicate plus 0.1). To normalize ODs between plates, a signal-to-cut off binding ratio (BR) was calculated for each sample by dividing sample OD by the cut-off value. A sample was considered reactive for all samples with a BR of ≥ 1.0.

#### Hybrid DABA

Total antibody to SARS-CoV-2 RBD was detected using the Imperial hybrid double antigen binding assay (DABA), Patent filing IRN.FID4816059. In this UKAS-accredited assay the solid-phase presentation of RBD is different from the RBD in the fluid phase. Microwells were coated with 100 µl of 2.5 µg/ml S1 antigen appropriately diluted in coating buffer (Clin-Tech) and incubated overnight at 2 to 8 °C. Wells were washed with PBS/0.05% Tween-20 once and blocked using 200 µl/well blocking solution (Microimmune) before drying overnight at 37 °C. Dried wells were stored desiccated at 4 °C. After allowing the solid phase to reach room temperature, 50 μl of serum and sample diluent or 100 µl of DBS eluate were added to each well and incubated for 1 h at 37 °C. Plates were washed five times (Wash buffer, Clin-Tech) followed by the addition of 100 µl of SARS-CoV-2 RBD antigen conjugated with HRP appropriately diluted in conjugate buffer (Clin-Tech). After a further incubation for 1 h at 37 °C, the plate was washed five times and 100 μl TMB substrate (Clin-Tech) added to each well, followed by a further 30 min incubation at 37 °C. Reactions were stopped by the addition of 50 μl/well 0.5 M sulphuric acid (Microimmune). The ODs were measured as described and BRs determined. A sample was considered reactive if it gave a BR of ≥ 1.0. This assay is 100% (95%CI 99.6–100%) specific, defined by testing 825 sera that pre-dated the epidemic, and 98.91% (96.8–99.8%) sensitive when evaluating 276 sera from individuals recovered from RT-PCR- confirmed SARS-CoV-2 infection^13,14^.

### Assay stability with imperfect sample collection

To investigate analytical stability, eluates from 11 sero-positive individuals were serially diluted twofold in elution buffer and the BR for each dilution from each individual were determined on the IgG S1 capture assay. Selected inoculated replicate blood spots were cut into half or quarter portions prior to extraction. Unused spots were cut into similar pieces and added appropriately to ensure the standard amount of matrix was included at the extraction step. Eluates produced from partial samples were intended to be comparable to those produced from the extraction of poorly inoculated partial spots normally considered invalid for use. Additionally, a DBS sample accidentally damaged whilst drying after collection was analysed.

### Study design for the validation of DBS testing

All serum samples were tested using Imperial Hybrid DABA to confirm sero status. Paired DBS samples were eluted and tested using IgG capture assay. Any IgG unreactive DBS sample paired with an anti-RBD positive serum were further tested using the IgM capture assay.

### Exploitation of DBS assay

#### Pilot sero-prevalence study

A total of 215 employees of a London-based law firm, at their request, carried out DBS tests at home by following the collection instructions described above. The tests were posted to the laboratory for antibody analysis as described.

#### Monitoring of post-vaccine responses

A small convenience sample (n = 34) of individuals living in London and the home counties who were early recipients of the first immunisation with either of the two currently MHRA approved vaccines^21,22^ requested to have their antibody responses tested by DBS. Participants were of ages ranging from 25 (health workers) to over 70 (first group to receive vaccine in the UK) and chosen at ≥ 14 days post-immunisation. All individuals were screened in order to exclude samples from anyone with a previous positive PCR test or who had experienced any of the classical symptoms associated with COVID within the preceding year.

### Statistical analysis

Data were analysed using Prism 8 software (GraphPad, San Diego, CA, USA). Sensitivity, specificity, positive and negative predictive value, and accuracy with 95% confidence interval of each assay to detect antibodies in DBS were calculated and compared.

Spearman’s test was used to determine if there was correlation between capture assay BR in paired DBS and serum samples. Wilcoxon signed-rank test was conducted to compare differences in BR between paired serum and plasma. Results were considered statistically significant if the *P* value was < 0.05. Wilcoxon matched pairs rank test was used to compare differences in BR in full spots, ½ spots and ¼ spots in DBS stability experiment. A DBS damaged after collection by a canine footprint was included in this analysis.

### Ethics

Serum and DBS samples taken under the REACT 2 study had ethical approval from South Central—Berkshire B Research Ethics Committee (REC ref: 20/SC/0206; IRAS 283,805). Samples for negative controls were taken from the Airwave study approved by North West—Haydock Research Ethics Committee (REC ref: 19/NW/0054). The study’s conduct and reporting are fully compliant with the World Medical Association’s Declaration of Helsinki on Ethical Principles for Medical Research Involving Human Subjects.

Serum and DBS samples for the development of the assays were also donated, following written, informed consent to the Communicable Diseases Research Tissue Bank of the Section of Virology (NRES ID 20/SC/0226). The use of these tissues was approved by the CDRTB Steering Committee in accordance with the responsibility delegated by the National Research Ethics Service.

All vaccine recipients sampled by DBS consented for their results to be used in this publication.

The corresponding author confirms that he had full access to all the data in the study and retains final responsibility for the decision to submit for publication.

### Patient and public involvement statement

Due to the urgency of the work and the current pandemic, it was not possible to confer with patient representatives. All ethical standards related to patient information were satisfied.

## Results

### Accuracy of DBS eluates on IgG and IgM S1 capture assays

**Figure 1:**
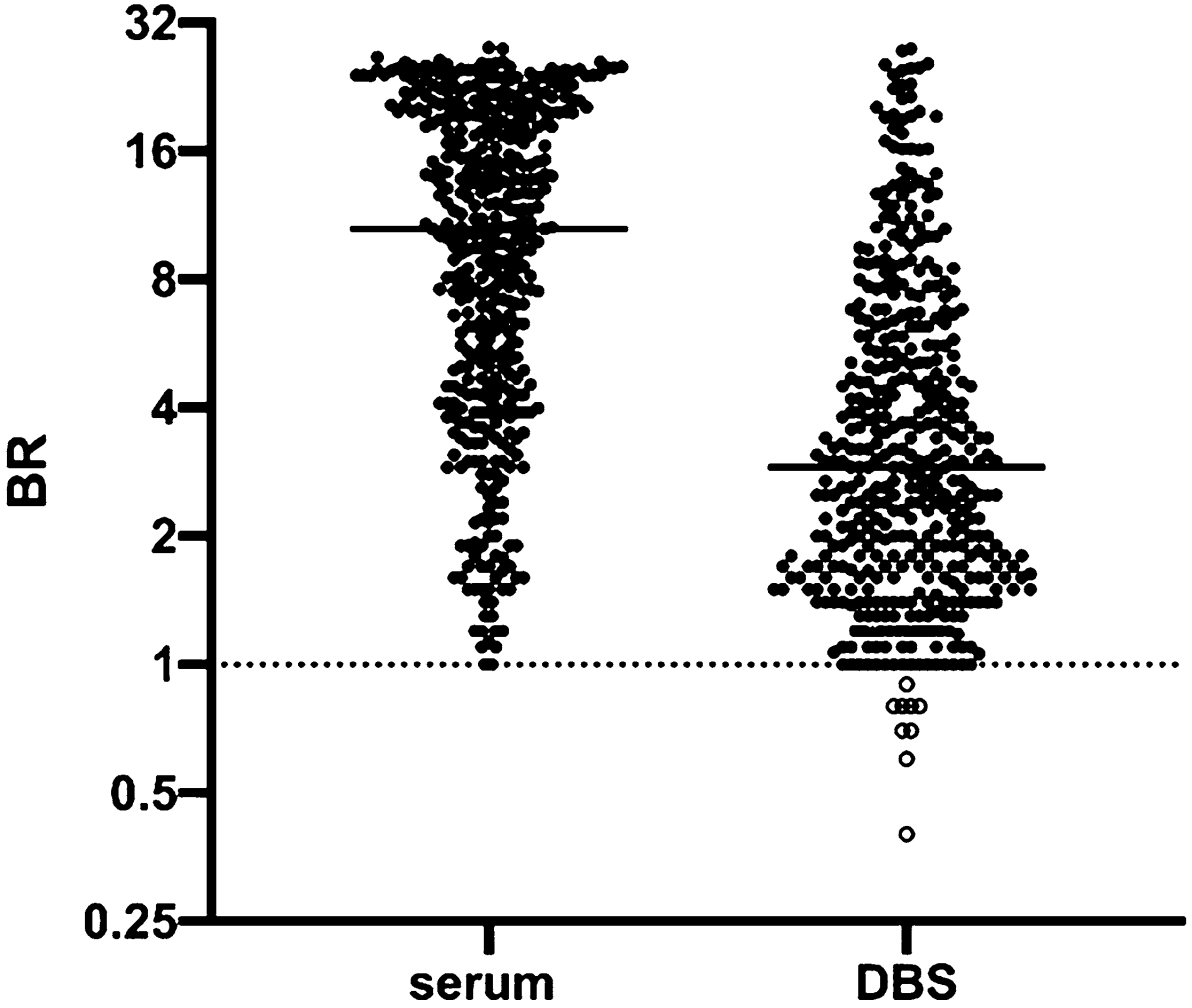
Comparison of Binding Ratios (BR) determined in Hybrid DABA assay using serum and Ig S1 capture assays using DBS eluates. Samples (serum) and DBS (eluates) from 439 sero-positive persons were assayed using the hybrid DABA and S1 IgG and IgM capture assays, respectively. Horizontal line indicates the mean. Binding ratios (BR) from both assays are plotted on a log2 scale with samples from sero-positive individuals shown with filled circles and those from sero-negative individuals shown with empty circles. The dotted line represents the assay cut-off. For 27 individuals, who were IgG negative but IgM positive on DBS eluate, IgM BR are used. Otherwise, IgG BRs are shown.

**Figure 2:**
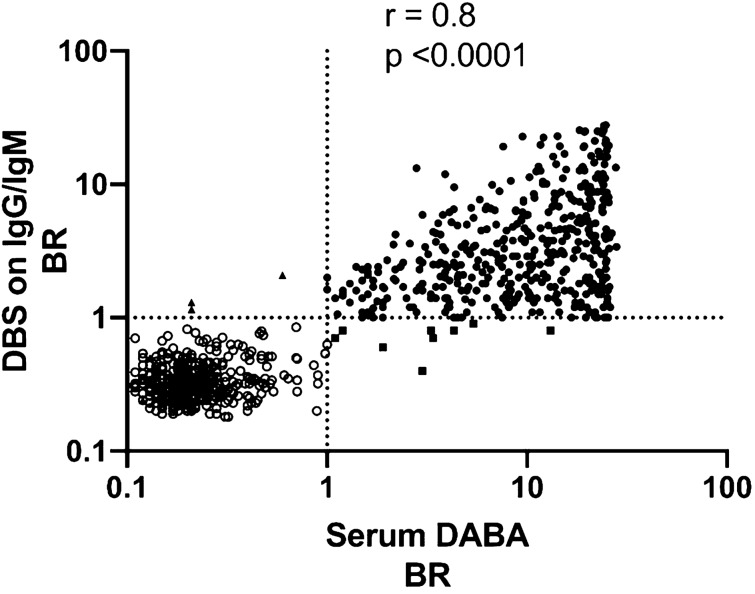
Correlation of BRs from S1 Ig assays using DBS eluates and Hybrid DABA assay using serum. Serum samples and paired DBS eluates from 439 sero-positive and 382 sero-negative individuals were assayed using hybrid DABA and the S1 IgG and IgM capture assays, respectively. BRs from DABA serum assay were plotted against those generated from the DBS eluate on a Log10 scale. Samples from sero-positive individuals are shown with filled circles and those from sero-negative individuals with empty circles. Correlation of the DBS and serum BRs with each assay is shown graphically along with the coefficient of correlation, r, and the significance, p, generated using PRISM software. The dotted lines represent the cut-off for each assay. Discordant samples, namely those that were DABA-positive and DBS-negative (n = 9) are represented by a square and samples which were DABA-negative and DBS-positive (n = 3) represented by a triangle.

Out of a total of 439 DBS samples from donors whose sera were reactive on the hybrid DABA, 430 were reactive in the capture assays (Fig. 1) when using IgG and IgM results combined, resulting in a diagnostic sensitivity of 97.9% (Table 1) and a positive predictive value of 99.3%. Of these 439 DBS eluates, 404 were reactive when tested for IgG antibody to S1, corresponding to a diagnostic sensitivity of 92% if using an IgG assay alone. The 26 which were unreactive for IgG antibody were reactive for IgM antibody to S1. Of the nine DBS samples which were unreactive in the capture assays, only one was linked with a serum reactive in the serum IgG and IgM capture assay (Supplementary Table 1). Detecting 430 of the 431 DBS samples linked with a parallel serum capture-reactive sample provides an analytical sensitivity of 99.7%. A correlation of 0.81 was observed when comparing BR from serum DABA and DBS IgG/IgM eluates (Fig. 2).

**Table 1:** Validation of DBS eluates on IgG and IgM S1 capture assays.

DBS donor characteristics	Number of DBS tested	Assay used for DBS	Number of DBS reactive	Measured outcome (95% CI)
DABA anti-RBD sero-positive	439	IgG and IgM**	430	Diagnostic sensitivity 97.9% (96.6–99.3)
		IgG alone	404	Diagnostic sensitivity 92.0% (89.5–94.6)
DABA anti-RBD sero-positive*	431	IgG and IgM**	430	Analytical sensitivity 99.8% (99.3–100)
DABA anti-RBD sero-negative	382	IgG	3	Diagnostic specificity 99.2% (98.4–100)

*This number excludes eight donors from the 439 donors whose sera were known to be negative by Ig Capture assay, ** IgG followed by IgM if IgG negative. Specificity and sensitivity shown are based on REACT donors many of whom will have been early convalescent patients.

Out of a total of 382 samples unreactive in the DABA, three eluates were reactive though of low BR in the Ig capture assay, giving a specificity of 99.2% (Table 1). The BR of these three samples which were sero-negative but DBS positive when assayed on S1 IgG and IgM capture are reported in Supplementary Table 1.

### Stability of capture BR on further dilution of DBS eluates and on partial or damaged sample eluates

**Figure 3:**
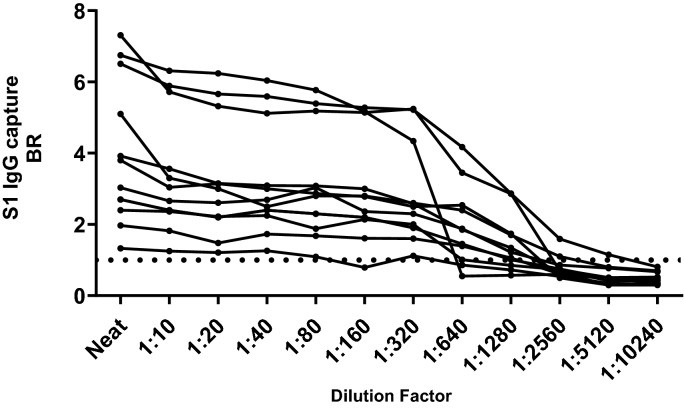
Analytical stability of DBS on IgG S1 capture assay. Eluates from eleven sero-positive DBS were serially diluted two-fold in elution buffer and the BRs determined on the IgG S1 capture assay. Each line with data points represents a different individual. The arrow indicates the highest dilution at which reactivity is maintained. The dotted line represents the assay cut-off.

**Figure 4:**
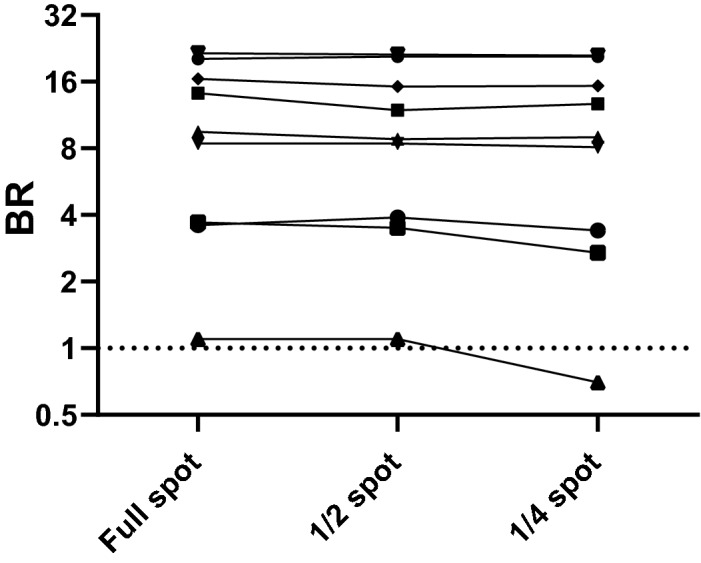
Reactivity of eluates on IgG S1 from full and partial DBS samples. DBS samples from 20 different patients were extracted as full, half and quarter spots as described in the methods. The reactivity of the three resulting eluates from each individual was tested in the IgG S1capture. The resulting reactivity (BRs) of the full, half and quarter spot eluates is shown for each individual, each line representing a different individual. The dotted line indicates the assay cut-off.

**Figure 5:**
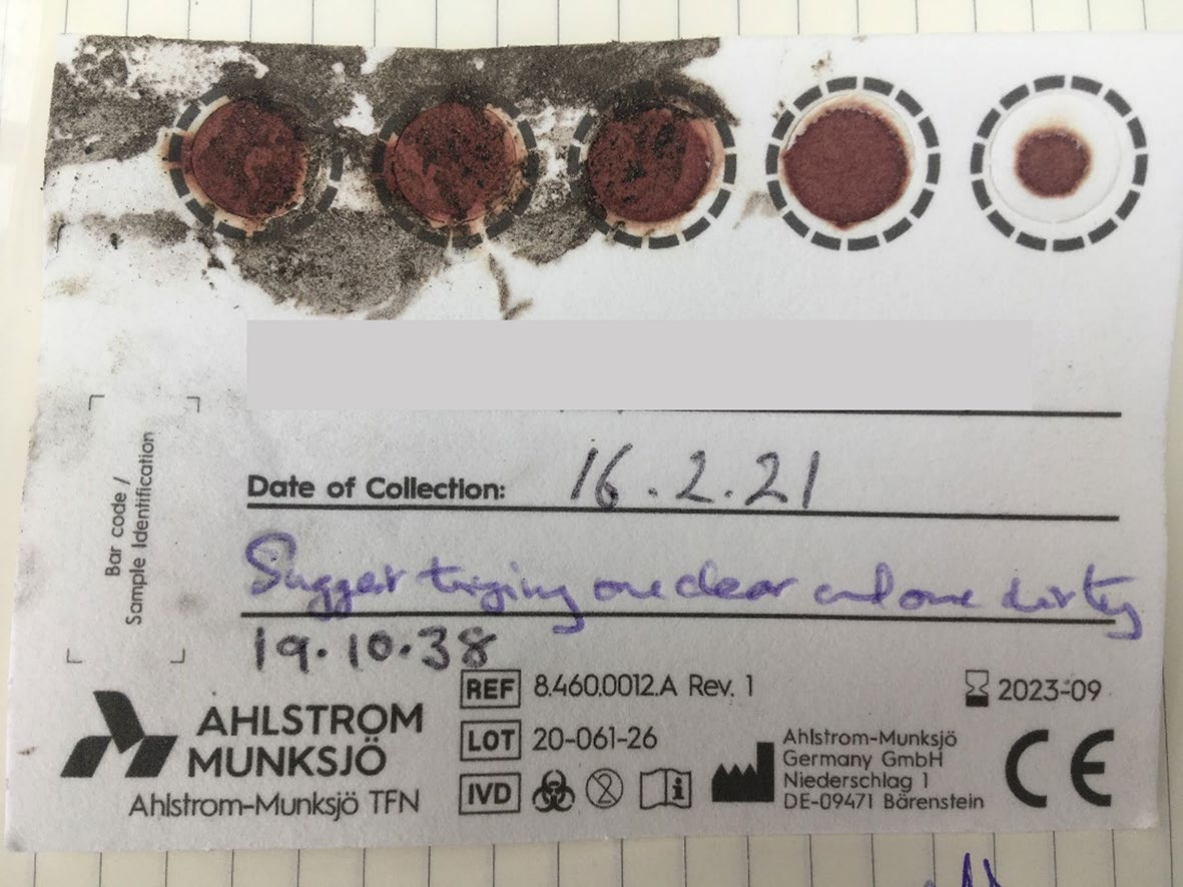
Impact of a wet and muddy canine foot upon a DBS from a recipient of a first dose of Pfizer vaccine. This exemplifies the damage accidently inflicted by a dog’s muddy paws on a DBS sample which had been laid out to dry. Eluates from the imprinted spots produced a valid strong positive Ig capture result.

Eleven DBS samples were subjected to serial dilution in the elution buffer which was free of human serum and then each dilution tested in S1 IgG capture. A plateau of reactivity was observed in all samples (Fig. 3). Twenty DBS samples were re-extracted as whole, half and quarter spots and the resulting eluates tested undiluted in the S1 IgG capture. Equivalent reactivity was seen in 19 sets of all samples (Fig. 4). No difference in reactivity was observed when comparing eluates from full spots and half spots (*p* = 0.276). A statistical difference was observed on comparing eluates from the half and quarter spots (*p* = 0.027) and when comparing the full spot and quarter spots (*p* = 0.039). Only one sample, with an initial BR of 1.2 when a full spot was tested, became undetectable with the quarter spot input. The accidentally damaged card (Fig. 5) yielded a valid eluate with a BR of 24.4.

### DBS performance in a pilot sero-prevalence study

**Figure 6:**
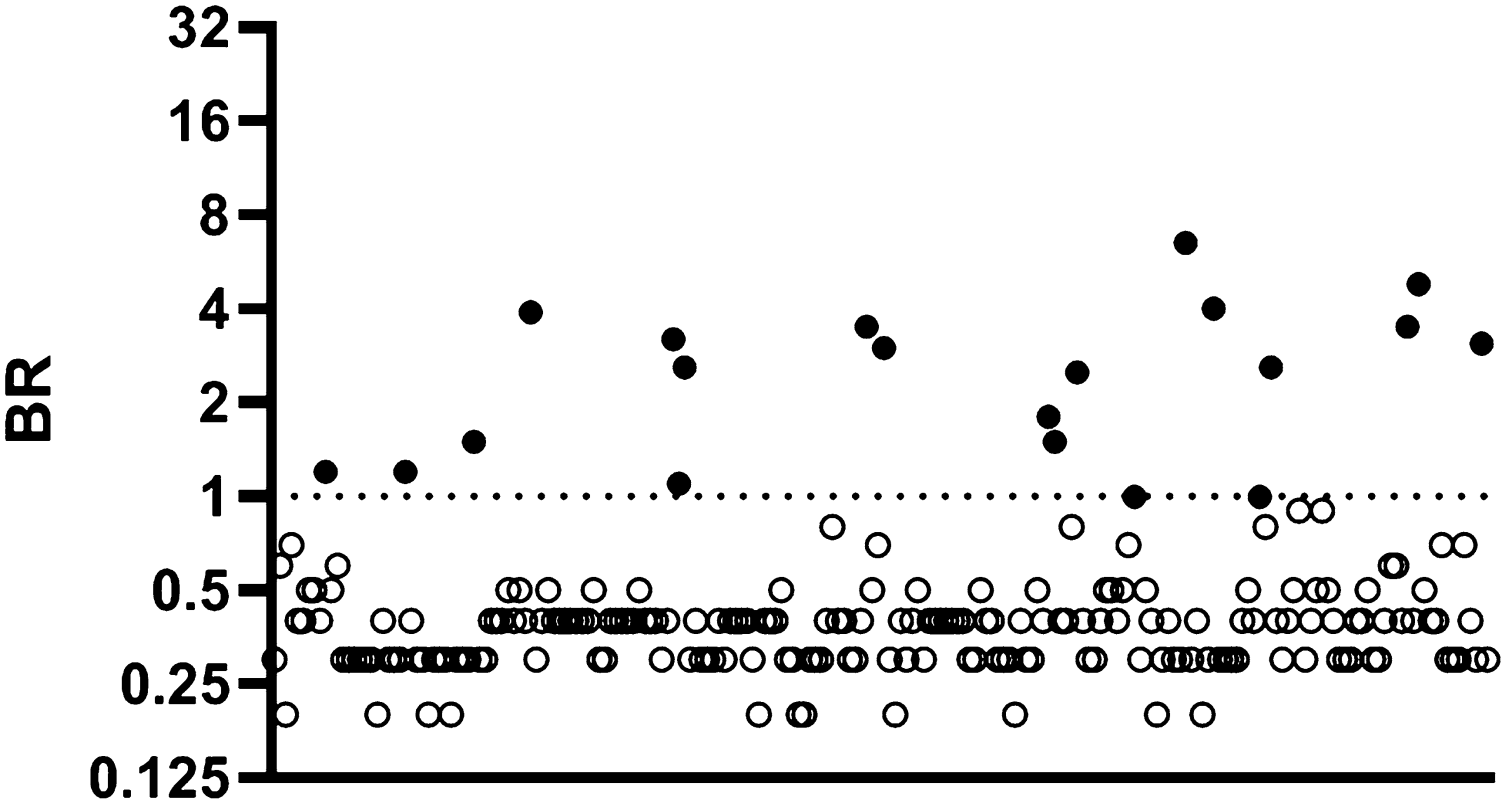
Seroprevalence determined by self-sampling and DBS analysis. DBS samples were obtained via self-sampling from 215 individuals employed in a London-based law firm. The BRs for S1 IgG alone are plotted on a log_2_ scale. Samples from 20 sero-positive individuals are shown with filled circles and those from sero-negative individuals with empty circles. The dotted line indicates the assay cut-off.

Samples from 20/215 individuals were found to be reactive for IgG antibody with a BR > 1 (Fig. 6). Two of these were on the cut-off above which samples are considered to be sero-positive (BR = 1). Four out of 20 reported mild to moderate symptoms, including loss of taste, smell, headache and cough. The remaining 16 individuals were asymptomatic.

### Humoral responses measured post-immunisation

**Figure 7:**
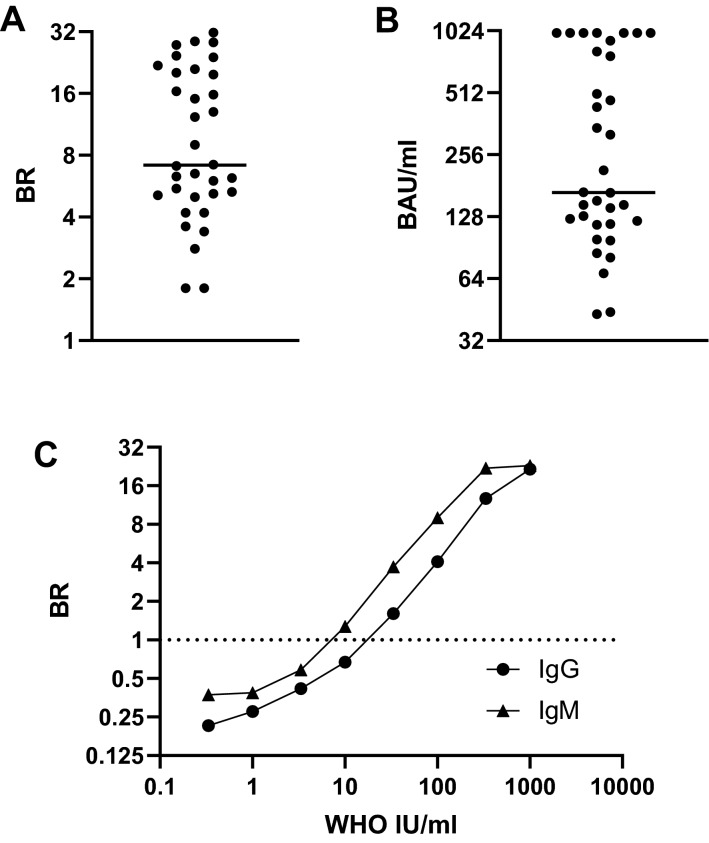
Measuring post-vaccine antibody responses using DBS sampling and the WHO International standard (NIBSC 20/136). DBS eluates obtained from 34 individuals who were ≥ 14 days post-immunisation are all reactive, demonstrating effectiveness at detecting vaccine-induced antibody responses. Binding Ratios (**A**) and inferred WHO International Units (**B**) from S1 IgG capture are displayed on a Log_2_ scale, the line is the median, BR of 1 and WHO32 IU are the cut off values. (**C**) The relationship between S1 IgM and IgG capture Binding Ratios and WHO IU/ml, with BR displayed on Log_2_ scale and IU/ml on a Log_10_ scale. IgG IU/ml are shown with a circle and IgM IU/ml are shown with a triangle.

A serologic response for IgG antibody was generated by all of the 34 sample eluates (Fig. 7) confirming the ability to detect vaccine-mediated antibody responses in this way.

### Performance of the NIBSC WHO International standard (Catalogue 20/136)

A half log_10_ series of dilutions of this standard in antibody-negative human plasma was constructed and run in both the IgM and the IgG capture assays (Fig. 7c). Greater potency was evident in the IgM assay. When titrated to extinction the lower limits for detection in the capture assays was equivalent to 23 WHO IU/ml in the IgG assay and 10 WHO IU/ml in the IgM assay.

## Discussion

In order to conduct large-scale sero-prevalence studies, a large number of rapid diagnostic tests targeting antibodies to SARS-CoV-2 were developed and are commercially available. A total of 249 are listed by the FIND foundation^23^. However, the performance of these assays remains inconsistent and does not meet the MHRA criteria for home testing of 98% sensitivity and 98% specificity^24,25^. Furthermore, home testing is not amenable to quality assurance. An alternative means of increasing testing capacity is to implement self-collection of diagnostic specimens, including the use of minimally invasively sampled capillary blood (DBS) with the samples being passed to suitably qualified reference laboratories. This sampling method has a high acceptance rate^26^. Here we have described and validated a highly sensitive and accurate immunoassay for the detection of antibodies to SARS-COV-2 in eluted DBS samples.

Although many may consider that testing DBS can be performed as an extension of existing analytical tests that use plasma or serum, it should be recognised that DBS eluates are likely to have a major impact on test performance, requiring a change in assay format. Indeed, the ensuing loss of sensitivity in such an exercise lead to publication of the belief that DBS sampling is inappropriate for determining SARS-CoV-2 prevalence^12^. The observation in our study where a DABA assay, as was the Roche assay in the aforementioned publication^12^, did not perform well with DBS was predictable (Supplementary Fig. 1). These assays, as well as indirect assays utilising labelled anti-Ig as detectors, require a high and predictable protein concentration in the first incubation. For the DABA, the absence of normal levels of serum protein in the first incubation would be expected to increase the off-rate during antibody binding, substantially reducing sensitivity as we observed. We note with interest the recent publication by Beyerl et al., who described a sensitivity of 99.2% using the Roche DABA assay, achieved by a substantial redesign of the assay cut-off for DBS eluates^27^.

To circumvent this requirement, we adapted and validated IgM and IgG capture assays that detect accurately antibodies to SARS-CoV-2 S1 in eluates from DBS (Fig. 1). The diagnostic sensitivity is 97.9%, but to achieve this requires testing for both IgG and IgM as the timing of the infection in relation to testing will not be known. The specificity of the capture assay (99.2%) using DBS is higher than that reported for other laboratory-based assays and validated using an adequate number of samples^15–17^. If a comparison is made excluding the DBS samples from those individuals whose sera were unreactive in the capture assay (Fig. 2), the analytical sensitivity becomes even greater (99.7%). The reactivity of IgG and IgM capture assays on DBS samples compared to that of parallel serum samples assayed on DABA also showed a strong correlation (r = 0.81). As the capture assays correlate with the DABA which in turn detects and measures anti-RBD, reactivity in the capture assays potentially predict the presence of neutralising antibody. The particular advantage offered by the capture immunoassay is that, providing there is sufficient total immunoglobulin to saturate the capturing antibody on the solid phase, the initial concentration of the target antibody in the analyte has limited effect on the sensitivity of the assay. Thus, the reactivity of a sample depends, not on absolute immunoglobulin titres, but on the proportion of the target antibody within the immunoglobulins captured by the anti-human antibody on the solid phase that is directed against the detector antigen, in this case S1.

This functionality is further exemplified by the observed plateau of reactivity, commensurate with saturation of the solid phase, observed when conducting serial dilutions in serum-free buffer of sample eluates in the IgG capture assay (Fig. 3). This is an important feature as it accommodates further sample dilution in serum-free fluids when eluting plasma proteins from the blood spot dried in the filter paper. Furthermore, there may be variations in the size of the blood spot collected and in the efficiency of elution, hence the amount of blood collected and Ig eluted, and consequently considerable variance of the final protein dilution factor. These are offset by the plateau of reactivity which lends robustness to the assay. The stability of signal on samples artificially-rendered invalid (Fig. 4) or on a damaged DBS sample (Fig. 5) further underlines this robustness. This is not a feature of other assay formats used in published assays that rely on adequately covered spots and either disqualify such incomplete or damaged samples or may have to rely on more than one spot to obtain valid results^15–17,28^.

DBS sampling is routinely used in the surveillance of a number of pathogens, such as HIV, Hepatitis B and Hepatitis C and is an important tool for seroprevalence studies, especially in those populations that are difficult to access^7,29^. We show that SARS-CoV-2 utilising S1 can be added to this list. We anticipate that a capture assay for NP antibody, currently under investigation, may further increase the utility of this approach and provide differentiation between virus induced antibody and vaccine responses.

We have proved the robustness of our assay in-field by conducting a sero-prevalence study in a London based law firm (Fig. 6) and believe that further such studies will play a vital role in encouraging a safe return to work when that time arises, on the evidence that sero-positivity from previous infection confers relative resistance to reinfection. We recognise that through only testing for IgG antibody we may have failed to detect a further two or three individuals. How common it may be in the recovered asymptomatic person representative of a community prevalence study to escape detection using only IgG antibody remains to be seen. The validation described is based on DBS samples from REACT study persons early in the convalescent period in whom a detectable IgG reactivity was yet to develop, accounting for the IgM Ig-capture-reactive samples. Steps will be undertaken to develop a protocol to circumvent this issue to avoid the need to use both an IgG and IgM assays should this be required. We are also confident that with the use of an appropriate assay, DBS sampling will greatly facilitate accurate and sensitive determination of seroprevalence.

The ability of this assay to detect vaccine-mediated antibody responses provides an essential resource during this pandemic (Fig. 7). It provides scientists with the opportunity to monitor and quantify in WHO international units the serological response to immunisation and, further, to examine the longevity of vaccine-mediated serological immunity using a minimally-invasive ‘at home’ methodology (Fig. 7b). We note, however, in this respect that the present International standard (NIBSC 20/136) is of greater IgM potency than IgG and sound a note of caution in this respect (Fig. 7c). We believe that the ability to use self-sampling coupled with an appropriate analytical process is not only essential to control the current pandemic, but will also provide an essential resource for future preparations. Our observations open up the real possibility of using self-sampling by DBS to allow sensitive, yet precise, measures of seroprevalence however this may have been generated. Finally, we would again emphasize that to do this, it is essential to use an antibody assay of the appropriate capture format and only by so doing will one facilitate population-based sero-epidemiological studies in the UK.

## Supplementary Information

Supplementary Information.
