# Supplementary material for: Simple, sensitive, specific self-sampling assay secures SARS-CoV-2 antibody signals in sero-prevalence and post-vaccine studies
Source: Sci Rep. 2022 Feb 3;12:1885. doi: 10.1038/s41598-022-05640-x (PMC8814240; doi:10.1038/s41598-022-05640-x)
Supplement: Supplementary file 1 — Supplementary Information. [file 41598_2022_5640_MOESM1_ESM.docx]

**Supplementary Msaterials**

**Simple, sensitive, specific self-sampling assay secures SARS-CoV-2 antibody signals in sero-prevalence and post-vaccine studies.**

**Authors**

Maryam Khan^1^, Carolina Rosadas^1^, Ksenia Katsanovskaja^1^, Isaac D Weber^1^, Justin Shute^2^, Samreen Ijaz^2^, Federica Marchesin^1^, Eleanor McClure^1^, Salem Elias^1^, Barnaby Flower^1^, He Gao, Rachael Quinlan^1^, Charlotte Short^1^, Annachiara Rosa^3^, Chloe Roustan^3^, Maya Moshe^1^, Graham P Taylor^1,4^, Paul Elliott^4,5,6^, Graham S Cooke^1,4^, Peter Cherepanov^3^, Eleanor Parker^1^, Myra O McClure^1^and Richard S Tedder* ^1^


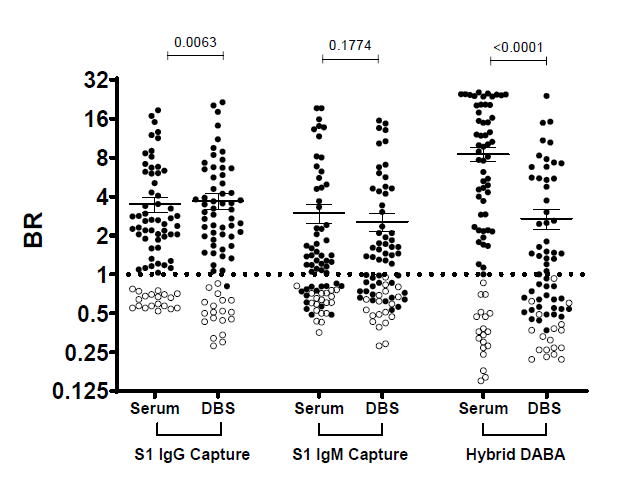


**Supplementary Figure 1. Comparison of Binding Ratios determined in Ig capture assays, hybrid DABA and DBS.** Samples (serum) and DBS (eluates) from 55 previously-infected persons were assayed in S1 IgG and IgM capture assays and in the hybrid DABA. Antibody status was compared when the same individuals’ sera and eluates were tested in the same assay. Binding ratios from all assays are plotted on a log2 scale with samples from sero-positive individuals shown with filled circles and those from sero-negative individuals shown with unfilled circles. The dotted line represents the cut-off values for the assays.

**Supplementary Table 1. Discordant reactivities displayed by sera and DBS eluates.**

| **Serum** | | | **DBS** | |
| --- | --- | --- | --- | --- |
| **DABA S/CO** | **S1 IgG S/CO** | **S1 IgM S/CO** | **S1 IgG S/CO** | **S1 IgM S/CO** |
| 5.4 | 2.6 | 1.0 | 0.4 | 0.6 |
| 1.2 | 0.6 | 0.7 | 0.7 | 0.7 |
|  |  |  |  |  |
| 1.9 | 0.4 | 0.6 | 0.7 | 0.7 |
| 1.5 | 0.4 | 0.5 | 0.6 | 0.6 |
| 1.2 | 0.6 | 0.7 | 0.8 | 0.7 |
| 1.8 | 0.4 | 0.6 | 0.8 | 0.5 |
|  |  |  |  |  |
| 4.2 | 0.5 | 0.7 | 0.8 | 0.8 |
| 2.5 | 0.9 | 0.6 | 0.9 | 0.5 |
| 9.2 | 0.5 | 0.5 | 0.8 | 0.7 |
| *0.6 | 1.9 | 0.2 | 2.10 | 0.4 |
| *0.2 | 1.05 | 0.3 | 1.12 | 0.5 |
| *0.2 | 1.09 | 0.3 | 1.11 | 0.3 |

Reactivity displayed by nine anti-RBD positive sera whose linked DBS samples were unreactive, the parallel sera tested in IgG and IgM S1 capture assays. *Reactivity displayed by three anti-RBD negative sera whose linked DBS samples were reactive, the parallel sera tested in IgG and IgM S1 capture assays.
